# The Value of Peripheral Blood Leukocyte Parameters in the Early Diagnosis and Clinical Prognosis of Sepsis

**DOI:** 10.1155/2023/6052085

**Published:** 2023-01-14

**Authors:** Yuandan He, Qianqian Liu, Lianhua Wei, Zhipeng Sun, Wenjuan Li, Fangmin Geng, Zhangping Lu, Hongwei Zhang

**Affiliations:** ^1^Department of Gansu University of Traditional Chinese Medicine, Lanzhou, Gansu, China; ^2^Department of Clinical Laboratory, Gansu Provincial Hospital, Lanzhou, Gansu, China; ^3^Department of Ningxia Medical University, Yinchuan, Ningxia, China; ^4^Department of Lanzhou University, Lanzhou, Gansu, China

## Abstract

**Background:**

Early diagnosis of sepsis is the key to timely, targeted treatment. Cell population data (CPD) has been widely used in many diseases, but its predictive value for early diagnosis and the clinical outcome of sepsis remains unclear. Therefore, this paper discusses whether peripheral blood leukocyte parameters can be used as predictive indicators for early diagnosis and the clinical outcome of sepsis.

**Methods:**

A retrospective study of 45 patients with sepsis, 53 patients with nonseptic infections, and 86 healthy check-ups admitted to Gansu Provincial Hospital from January 2021 to June 2022 was done using a hematology analyzer.

**Results:**

The results of LYMPH#, HFLC#, IG#, NE-WX, LY-WX, LY-WY, and MO-WX showed better diagnostic efficiency in the sepsis group and nonseptic infection group. When the seven differential leukocyte parameters were used to establish diagnostic models, the sensitivity and specificity were 82.20% and 77.40%, respectively. Correlation analysis showed that LYMPH# and HFLC# were positively correlated with PCT (*P* < 0.05). The clinical outcome of sepsis showed that the leukocyte parameters of discharged WBC and LY-X had better predictive efficacy. When the two differential leukocyte parameters were used to establish diagnostic models, the sensitivity and specificity were 90.90% and 100.00%. Cox regression analysis showed that leukocyte parameters of discharged WBC and LY-X were independent predictors of clinical outcomes (*P* < 0.05).

**Conclusion:**

Leucocyte parameters HFLC#, IG#, NE-WX, LY-WX, LY-WY, and MO-WX had a certain auxiliary effect on the early diagnosis of sepsis leukocyte parameters of discharged WBC and LY-X were independent predictors of clinical outcomes in patients with sepsis. Therefore, peripheral blood leukocyte parameters may have predictive value for early diagnosis and the clinical outcome of sepsis, but large-scale retrospective studies are still needed to prove our preliminary results.

## 1. Introduction

Sepsis is a life-threatening systemic inflammatory response syndrome with organ dysfunction caused by the dysregulated host response to infection [1]. Sepsis has a high morbidity and mortality rate and a very poor prognosis, and the incidence tends to increase year by year, causing a serious social burden. Therefore, sepsis has become an important global public health problem [2–4]. In the past few decades, a large number of serum (plasma) experimental tests have been conducted on sepsis patients, and the molecular markers of sepsis have been found to include C-reactive protein (CRP), procalcitonin (PCT), presepsin, interleukin-6 (IL-6), and neutrophil CD64 [5–7]. During the study, it was found that PCT and presepsin may be the most effective detection means for early diagnosis, prognostic monitoring, and clinical treatment of sepsis [8, 9]. However, it has not been fully verified that these biomarkers can help clinicians identify sepsis as early as possible and accurately, carry out treatment, and predict prognosis [10, 11]. As a result, interest in identifying new, low-cost, routinely available indicators of infection has been stimulated. Studies have shown [12–14] that changes in the response of cell population data (CPD) to various stimuli (such as infection) can rapidly provide information on leukocyte activation, such as the cell complexity, fluorescence intensity, cell size, and distribution width of neutrophils, monocytes, and lymphocytes, which can quantitatively analyze cell morphology and function. At the same time, the method of acquisition and operation is simple, convenient, and rapid, which provides a new method to improve the early diagnosis of sepsis. Notably, the new generation of hematology analyzers can automatically obtain CPD parameters during standard cell count analysis, significantly reducing the need for additional blood tests and costs [15]. The aim of this study was to evaluate the clinical relevance of leukocyte parameters as early diagnostic parameters of sepsis or septic shock and to test the predictive role of leukocyte parameters in the prognosis of sepsis.

## 2. Materials and Methods

### 2.1. General Information

A total of 45 patients with sepsis and 53 patients with nonseptic infections who visited Gansu Provincial Hospital from January 2021 to June 2022 were selected as research subjects. Another 86 healthy subjects underwent physical examination in the same period and were selected as the healthy control group. Laboratory and auxiliary examination results and clinical outcomes of patients with sepsis were collected.

#### 2.1.1. Inclusion Criteria

In line with the definition of sepsis in the “Save Sepsis Movement: Guidelines for the International Management of Sepsis and Septic Shock (2016)” jointly developed by the American Society of Critical Care Medicine and the European Society of Critical Care Medicine in 2016 [1].

#### 2.1.2. Exclusion Criteria

(1) Do not actively cooperate or give up halfway; (2) incomplete case data after admission; (3) admission time <24 hours; (4) severe liver and kidney diseases, advanced malignant tumors, hematological diseases, serious heart diseases, and acute cerebrovascular diseases.

### 2.2. Study Groups

1. According to the definition of sepsis [1], the patients were divided into the sepsis group, nonseptic infection group, and healthy control group.
2. According to the clinical outcome, the patients were divided into an unhealed group and a cured group.

### 2.3. Study Methods

Blood culture and bacterial identification were performed using the BacT/Alert3D blood culture instrument and the VitEK-2 automatic bacterial identification system. Blood samples were collected for peripheral blood cell analysis using a hematology analyzer (Sysmex XN9000®) and matching reagents. 26 leukocyte parameters were recorded in the sepsis group, the nonseptic infection group, the healthy control group at admission (*t*0), and the sepsis group at discharge.

### 2.4. Primary Outcome

We evaluate the predictive value of peripheral blood leukocyte parameters for early diagnosis and the clinical outcome of sepsis.

### 2.5. Secondary Outcomes

We investigate whether there is a correlation between peripheral blood leukocyte parameters and PCT in the early stage of sepsis and the diagnostic value of PCT in the clinical outcome analysis of sepsis patients.

### 2.6. Ethics

The study was approved by the Institutional Ethics Committee of Gansu Provincial Hospital. Written informed consent was obtained from each participant or their family members.

### 2.7. Statistical Analysis

PASS11 software was used to estimate the sample size: this study was a randomized controlled trial designed in parallel, and the two groups were the sepsis and nonseptic infection groups, respectively. The peripheral blood leukocyte parameter values of the study subjects were the main observational outcome index. According to previous literature reports (or pretest results), NA = 37 cases and NB = 37 cases were calculated. Assuming that the loss of follow-up rate of the subjects is 10%, sample size NA = 37 ÷ 0.9 = 41 cases, NB = 37 ÷ 0.9 = 41 cases. Finally, 41 cases were included in the sepsis group, and 41 cases were included in the nonseptic infection group, for a total of at least 82 cases.

Statistical analysis was conducted using IBM SPSS STATISTICS (version 26.0). Normal distribution measurement data were expressed as *x* ± *s*, analysis of variance was compared between groups, non-normal distribution measurement data were expressed as *M* (P25, P75), and a nonparametric rank sum test wad compared between groups. Pearson's correlation analysis was performed between PCT and leukocyte parameters. Multivariate Cox regression analysis was used to determine the risk factors. The receiver operating characteristic (ROC) curve and the area under the curve (AUC) were drawn to evaluate the differential diagnostic efficacy of leukocyte parameters and the predictive value of clinical outcome. *P* < 0.05 indicated statistical significance.

## 3. Results

  (3.1) Brief description of leukocyte parameters [16]. Please see Table 1
  (3.2) Diagnostic value of peripheral blood leukocyte parameters in early sepsis

### 3.1. Comparison of General Clinical Data between the Sepsis Group and Nonseptic Group

There were no significant differences in gender, age, underlying diseases, and multiple site infection between the sepsis and nonseptic groups (*P* > 0.05). The pulse and maximum body temperature of the sepsis group were higher than those of the nonseptic group, and the systolic blood pressure and diastolic pressure were significantly lower than those of the nonseptic group (*P* < 0.05), Table 1, as shown in Table 2.

### 3.2. Comparison of Leukocyte Parameters between the Sepsis Group, Nonseptic Infection Group, and Healthy Control Group

WBC, NEUT#, LYMPH#, MONO#, EO#, HFLC#, IG#, NE-SFL, LY-Y, LY-Z, MO-X, MO-Y, MO-Z, NE-WY, NE-WZ, LY-WX, LY-WZ, MO-WX, MO-WY of sepsis group and the nonseptic infection group were compared with the healthy control group, and the difference was statistically significant (*P* < 0.05). LYMPH#, BASO#, HFLC#, IG#, NE-WX, LY-WX, LY-WY, MO-WX of the sepsis group were higher than those of the nonseptic infection group, and NE-FSC of the sepsis group was lower than that of the nonseptic infection group, and the differences were statistically significant (*P* < 0.05). Please see Table 3.

### 3.3. Efficacy Evaluation of Leukocyte Parameters in Differential Diagnosis between Septic and Nonseptic Infection Groups

**Figure 1:**
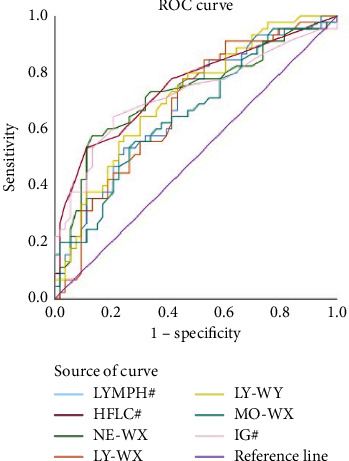
ROC curve for differential diagnosis of leukocyte parameters between sepsis and nonseptic infection.

**Figure 2:**
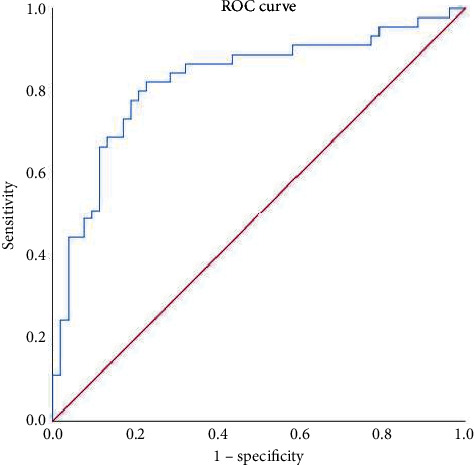
ROC curve drawn by LYMPH#, HFLC#, IG#, NE-WX, LY-WX, LY-WY, and MO-WX.

LYMPH#, HFLC#, IG#, NE-FSC, NE-WX, LY-WX, LY-WY, MO-WX with statistically significant leukocyte parameters were selected to make ROC curves for differential diagnosis of sepsis and nonseptic infection, and LYMPH#, HFLC#, IG#, NE-WX, LY-WX, LY-WY, MO-WX, and the area under the curve >0.60, have a better differential diagnosis performance in early sepsis, as shown in Figure 1. Seven differential leukocyte parameters were used to establish diagnostic models, as shown in Figure 2. Leukocyte parameters such as AUC, cut-off, sensitivity, specificity, positive predictive value, and negative predictive value are as shown in Table 4.

### 3.4. The Correlation between Leukocyte Parameter Results and PCT in Sepsis and Non-Septic Infection

**Figure 3:**
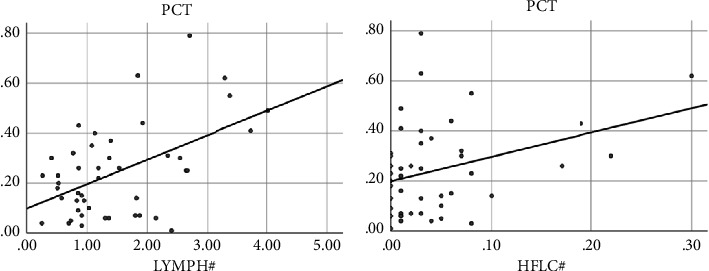
Correlation between leukocyte parameter and PCT.

LYMPH#, HFLC#, and PCT were positively correlated (*P* < 0.05) as shown in Table 5. The correlation analysis between LYMPH#, HFLC#, and PCT is shown in Figure 3.

### 3.5. Predictive Value of Peripheral Blood Leukocyte Parameters for Clinical Outcomes of Patients with Sepsis

#### 3.5.1. Comparison of Leukocyte Parameters between Unhealed and Cured Groups

The results showed that three of the 26 leukocyte parameters were statistically significant in the clinical outcome analysis of sepsis patients (*P* < 0.05), which were WBC, NEUT#, and LY-X as shown in Table 6.

#### 3.5.2. Influence of Leukocyte Parameters on Clinical Outcomes of Patients with Sepsis

Univariate Cox regression analysis showed that WBC, NEUT#, and LY-X during admission and discharge were predictive factors of clinical outcome in patients with sepsis (*P* < 0.05). Multivariate Cox regression analysis showed that WBC and LY-X at discharge were still independent predictors of clinical outcome in patients with sepsis (*P* < 0.05), as shown in Table 7.

#### 3.5.3. Predictive Value of Leukocyte Parameters and PCT for Clinical Outcome in Patients with Sepsis

**Figure 4:**
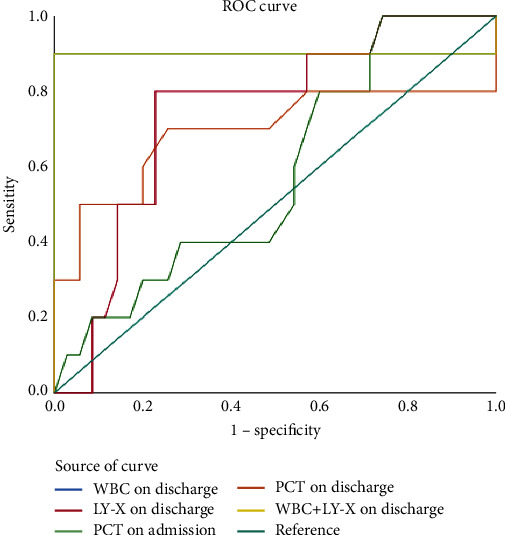
ROC curve of leukocyte parameters and PCT predicting clinical outcome of sepsis patients.

The results of ROC analysis showed that the discharge leukocyte parameters WBC and LY-X had better performance in predicting the clinical outcome of patients with sepsis. Combined diagnosis of discharged leukocyte parameters WBC and LY-X, as shown in Figure 4. Leukocyte parameters such as AUC, cut-off, sensitivity, specificity, positive predictive value, and negative predictive value are as shown in Table 8.

## 4. Discussion

Each year millions of patients died of sepsis, mortality rate close to 30%, which caused serious damage to human health, so the early recognition and appropriate treatment is crucial for improving the prognosis of patients with sepsis, but the general blood culture and drug sensitive test need 3–5 d out as a result, so early identification of bacterial infections or suspicions is the first step toward sepsis treatment [17]. Therefore, it is particularly important to provide clinically objective, rapid, and accurate experimental detection indicators for the diagnosis and symptomatic treatment of sepsis patients. In this study, we analyzed the parameters of peripheral blood leukocytes in patients with sepsis to explore their diagnostic value for sepsis. Related literature [18–20] has shown that when the body is infected, the changes of peripheral blood cells are not only the single occurrence of WBC and the proportional change of various classification counts, but also the generation of rod-shaped nuclei and other immature granulocytes and the morphological changes of numerous cells, including the appearance of neutrophils toxic particles, vacuoles, and dule bodies. The cytoplasmic particles of lymphocytes increased, and their volume increased. Monocytes migrate and deform, and their volume and morphology change to some extent. At the same time, through the analysis of the indicators reflecting the left shift in granulocyte, monocyte, and lymphocyte morphology and the change in intracytoplasmic structure complexity, it was found that they have a certain value in predicting and differentiating infection.

It was found that the number of lymphatic markers HFLC#, IG#, NE-WX, LY-WX, LY-WY, and MO-WX was significantly changed in the differential diagnosis of the sepsis group and the nonseptic infection group. Lymphatic #, BASO#, HFLC#, IG#, NE-WX, LY-WX, LY-WY, and MO-WX were higher than those of the nonseptic infection group and could be correlated with the increase of peripheral blood mononuclear cells and neutrophil and lymphocyte cytoplasmic particles caused by the activation of the mononuclear macrophage system after infection, while NE-FSC was lower than that of the sepsis group. In the nonseptic infection group, immunosuppression may be associated with the immune imbalance in the body when severe bloodstream infection occurs, which further confirmed the changes in the size and internal structure of neutrophils and lymphocytes in the development of sepsis patients [13, 14, 21], and the combined diagnosis of these indicators was more effective. Its sensitivity and negative predictive value were higher than those of a single test. HFLC# is a new quantitative parameter provided by a blood cell analyzer based on the principle of flow cytometry combined with nucleic acid fluorescence staining technology. It can detect the quantitative indicators of lymphocyte qualitative change from different angles and does not depend on the quantity change. The results of this study showed that the specificity and positive predictive value of HFLC# in the sepsis group were higher than other monitoring indicators, and the value (reference range of HFLC#: 0-0.01 Gpt/l) was higher than that of the nonseptic infection group and the healthy body test group, which was consistent with the research results of Arneth et al. [22]. Related literature also shows that HFLC# has been intensively studied as a potential marker of sepsis [23, 24].

In the clinical outcome analysis of patients with sepsis, the leukocyte parameters WBC and LY-X in discharge were independent risk factors for predicting the clinical outcome of patients with sepsis. When the body is infected and tissue is damaged, WBC will rapidly increase its accumulation and phagocytose the invading pathogens [25]. As a routine clinical examination indicator, WBC plays a certain guiding role in the body infection. However, due to the large individual differences and the fact that WBC is easily affected by mental, emotional, sports, and surrounding environment factors, its normal range value is relatively wide, which has certain limitations in the diagnosis of sepsis patients [26]. Therefore, it is often necessary to combine it with other indicators to make a more accurate judgment of the patient's condition. Therefore, the sensitivity, specificity, positive predictive value, and negative predictive value of the combined diagnosis of the discharged leukocyte parameters WBC and LY-X have better predictive value for the clinical outcome of patients with sepsis.

As a precursor of calcitonin, PCT is produced by the thyroid gland when the body is not infected. When the body has a severe systemic infection, the lung, liver, kidney, brain, and pancreas are the main sources of PCT [27]. The value of procalcitonin is positively correlated with the severity of infection and is one of the most commonly used inflammatory indicators in clinical practice [28]. The value of procalcitonin can increase rapidly in 2∼4 h after infection, and reaches a peak in 24∼48 h, and can increase to 1000 times of the normal value in severe infection. A number of studies have shown that CRP, IL-6, and other markers have more diagnostic value in sepsis [29, 30]. There was a correlation between LYMPH# and HFLC# and the inflammatory index PCT in the differential diagnosis of sepsis group and nonseptic infection group, but the correlation was weak. In addition, in the clinical outcome analysis of patients with sepsis, the predictive ability of PCT was weaker than that of the combined diagnosis of WBC and LY-X in discharge. The main reasons may be two aspects: First, the variation of serum PCT levels in sepsis patients is large, and PCT is often difficult to accurately reflect the occurrence and progression of sepsis at the early stage of the disease. Secondly, in different stages of disease, differences in detection methods, size of infected organs, types of pathogenic bacteria, and the immune inflammatory state of the body make it difficult to uniformly define the critical value of PCT [31]. Traumatic stress and surgery can also cause an increase of the serum PCT level [32]. However, the results of this study enhance the predictive value of peripheral blood leukocyte parameters for sepsis, so clinicians can consider it an auxiliary indicator. However, the results of this study point out the predictive value of peripheral blood leukocyte parameters for sepsis, so clinicians can consider it an auxiliary indicator.

In summary, peripheral blood leukocyte parameters may be helpful for clinicians to predict early diagnosis and the clinical outcome of sepsis. However, this study has certain limitations: (1) The included study is a retrospective clinical study, and selection bias is inevitable. We will conduct a larger prospective study in the future; (2) the sample size of some included studies is relatively small, which may lead to the bias of the analysis results; and (3) patients with sepsis were not graded for severity. Despite these limitations, our study provides new insights into the value of peripheral blood leukocyte parameters in the early diagnosis and clinical outcome of sepsis.

## Figures and Tables

**Table 1:** Brief description of leukocyte parameters.

Leukocyte parameters	Cell type description and instructions
WBC	White blood cell count
NEUT#	Neutrophil absolute concentration
LYMPH#	Lymphocyte absolute concentration
MONO#	Monocyte absolute concentration
EO#	Eosinophil absolute concentration
BASO#	Basophil absolute concentration
HFLC#	High fluorescence large cell absolute concentration
IG#	Immature granulocyte absolute concentration
NE-SSC	Mean side scattered light distribution width of the neutrophil
NE-SFL	Mean fluorescent light distribution width of the neutrophil
NE-FSC	Mean forward scattered light distribution width of the neutrophil
LY-X	Mean side scattered light intensity of the lymphocyte
LY-Y	Mean fluorescent light intensity of the lymphocyte
LY-Z	Mean forward scattered light intensity of the lymphocyte
MO-X	Mean side scattered light intensity of the monocyte
MO-Y	Mean fluorescent light intensity of the monocyte
MO-Z	Mean forward scattered light intensity of the monocyte
NE-WX	Side scattered light distribution width of the neutrophil
NE-WY	Fluorescent light distribution width of the neutrophil
NE-WZ	Forward scattered light distribution width of the neutrophil
LY-WX	Side scattered light intensity of the lymphocyte
LY-WY	Forward scattered light intensity of the lymphocyte
LY-WZ	Forward scattered light intensity of the lymphocyte
MO-WX	Side scattered light distribution width of the monocyte
MO-WY	Fluorescent light distribution width of the monocyte
MO-WZ	Forward scattered light distribution width of the monocyte

**Table 2:** Comparison of general clinical data between sepsis and nonseptic infection groups.

Clinical features	Sepsis group (*n* = 45)	Nonseptic infection (*n* = 53)	*t*-value	*P* value
Gender (male/female)	23/22	32/21	61.99	0.357
Age	57.5 ± 12.68	52.79 ± 10.59	3.47	0.601
Underlying diseases				
Hypertension	11.00 (24.44)	9.00 (16.98)	78.38	0.503
Diabetes	6.00 (13.33)	5.00 (9.43)	80.79	0.234
Maximum body temperature (°C)	37.05 ± 0.35	36.90 ± 0.19	2.58	0.012
Multiple site infection	8.00 (17.78)	6.00 (11.32)	71.90	0.001
Pulse (times/min)	99.11 ± 10.26	89.49 ± 5.31	5.68	0.286
Systolic blood pressure (mm Hg)	118.58 ± 9.42	122.13 ± 8.16	−2.00	0.048
Diastolic blood pressure (mm Hg)	66.13 ± 5.76	74.47 ± 4.34	−7.98	0.001

Abbreviations: 1 mm Hg = 0.133 kPa.

**Table 3:** Leukocyte parameter test results of sepsis group, nonseptic infection group, and healthy physical examination group.

Leukocyte parameters	Sepsis group (*n* = 45)	Non-septic infection group (*n* = 45)	Healthy control group (*n* = 86)	*P* value
WBC (×10^9^/L)	9.62 (7.13, 14.42)	8.80 (6.88, 10.80)	5.90 (4.95, 6.80)	<0.05^bc^
NEUT# (×10^9^/L)	7.56 (5.51, 11.94)	7.12 (5.37, 9.21)	3.22 (2.54, 4.01)	<0.05^bc^
LYMPH# (×10^9^/L)	1.49 ± 0.95	0.96 ± 0.71	2.03 ± 0.47	<0.05^abc^
MONO# (×10^9^/L)	0.58 (0.46, 0.75)	0.53 (0.35, 0.70)	0.39 (0.33, 0.47)	<0.05^bc^
EO# (×10^9^/L)	0.02 (0.00, 0.10)	0.01 (0.00, 0.04)	0.09 (0.06, 0.15)	<0.05^bc^
BASO# (×10^9^/L)	0.02 (0.01, 0.02)	0.01 (0.00, 0.01)	0.02 (0.02, 0.03)	<0.05^ac^
HFLC# (×10^9^/L)	0.03 (0.01, 0.06)	0.00 (0.00, 0.01)	0.00 (0.00, 0.00)	<0.05^abc^
IG# (×10^9^/L)	0.11 (0.55, 0.335)	0.05 (0.03, 0.08)	0.01 (0.01, 0.02)	<0.05^abc^
NE-SSC	147.33 ± 8.56	150.76 ± 4.91	150.15 ± 3.00	—
NE-SFL	50.90 (47.90, 58.85)	49.90 (45.8, 53.75)	41.40 (40.18, 43.13)	<0.05^bc^
NE-FSC	82.34 ± 8.44	89.03 ± 4.8	85.00 ± 2.68	<0.05^ac^
LY-X	77.91 ± 4.51	77.23 ± 4.01	76.08 ± 1.29	—
LY-Y	67.30 (62.85, 72.50)	65.10 (60.95, 70.15)	57.70 (56.30, 59.43)	<0.05^bc^
LY-Z	58.00 (55.85, 60.30)	58.80 (57.10, 59.90)	54.25 (53.80, 55.00)	<0.05^bc^
MO-X	119.50 (117.45, 122.35)	118.20 (114.70, 121.20)	113.85 (113.20, 114.70)	<0.05^bc^
MO-Y	115.40 (106.45, 124.15)	113.20 (105.4, 118.1)	97.65 (94.8, 101.23)	<0.05
MO-Z	66.94 ± 3.86	67.86 ± 3.17	63.51 ± 1.79	<0.05^bc^
NE-WX	337 (316.5, 361.5)	316 (304.50, 325)	308.50 (298, 318)	<0.05^ab^
NE-WY	671 (619, 791.5)	698 (662.5, 773.5)	611.50 (598, 628)	<0.05^bc^
NE-WZ	738 (706.5, 770)	703 (680.5, 730.5)	638.5 (621.75, 661)	<0.05^bc^
LY-WX	554 (510, 630.5)	501 (457, 567)	461 (440, 489.5)	<0.05^abc^
LY-WY	1010 (913.5, 1102.5)	902 (814, 996)	870.5 (832.25, 919.5)	<0.05^ab^
LY-WZ	624 (574.5, 716.5)	622 (576.5, 658.5)	572 (559, 593.75)	<0.05^bc^
MO-WX	277 (247, 296)	253 (234, 279)	243 (230.75, 253)	<0.05^abc^
MO-WY	727 (668.5, 799.5)	707 (643, 773)	649.5 (597.25, 708.25)	<0.05^bc^
MO-WZ	665.56 ± 150.17	630.55 ± 85.34	617.20 ± 68.85	—

Note: the letters in superscript indicate the results of post hoc tests: ^a^significant difference between the septic and nonseptic infection groups in post hoc comparison; ^b^significant difference between the septic and healthy control groups in post hoc comparison; ^c^significant difference between the nonseptic infection and healthy control groups in post hoc comparison.

**Table 4:** Efficacy evaluation of leukocyte parameters in differential diagnosis between sepsis and nonseptic infection.

Leukocyte parameters	AUC (95%CI)	Cut-off	Se (%)	Sp (%)	PPV (%)	NPV (%)
LYMPH#	0.686 (0.580, 0.791)	0.84	75.60	56.60	59.67	73.21
HFLC#	0.751 (0.652, 0.849)	0.03	53.30	88.70	80.02	69.11
NE-WX	0.722 (0.617, 0.827)	331.50	57.80	86.80	78.80	70.78
LY-WX	0.677 (0.571, 0.784)	506.50	77.80	54.70	59.32	74.37
LY-WY	0.713 (0.612, 0.814)	954.00	64.40	69.80	64.42	69.78
MO-WX	0.657 (0.549, 0.765)	276.50	55.60	71.70	62.52	65.54
IG#	0.724 (0.619, 0.829)	0.09	64.40	79.20	72.44	72.38
LYMPH# + HFLC# + NE-WX + LY-WX + LY-WY + MO-WX + IG#	0.829 (0.739, 0.913)	0.62	82.20	77.40	75.54	83.66

Abbreviations. AUC, area under the receiver operating characteristic curve; CI, confidence interval; Se, sensitivity, Sp, specificity; PPV, positive pretest value; NPV, negative pretest value.

**Table 5:** Correlation between leukocyte parameter and PCT (*R*).

Indicators	LYMPH#	HFLC#	IG#	NE-WX	LY-WX	LY-WY	MO-WX
PCT	0.515	0.339	0.083	0.006	−0.043	0.037	−0.097

**Table 6:** Detection results of leukocyte parameters in admission group and discharge group.

Leukocyte parameters	Unhealed group (*N* = 45)	Cured group (*N* = 45)	*F*/*t*	*P* value
Leukocyte parameters on admission				
WBC (×10^9^/L)	19.0 (15.4, 21.0)	8.7 (6.9, 11.8)	−3.6	<0.05
NEUT# (×10^9^/L)	16.2 (13.1, 19.3)	6.9 (5.0, 7.9)	−3.6	<0.05
LY-X	75.4 ± 5.4	78.7 ± 4.0	2.1	<0.05
Leukocyte parameters on discharged				
WBC (×10^9^/L)	11.3 (10.9, 11.9)	5.5 (4.0, 7.9)	−3.8	<0.05
NEUT# (×10^9^/L)	9.1 (9.0, 9.6)	3.8 (3.0, 5.1)	−3.8	<0.05
LY-X	77.1 (74.5, 78.6)	78.6 (76.3, 81.0)	−2.3	<0.05

**Table 7:** Cox regression analysis of leukocyte parameters on clinical outcome of sepsis patients.

Leukocyte parameters	Univariate cox regression		Multivariate cox regression analysis	
	HR	95% CI	HR	95% CI
Leukocyte parameters on admission				
WBC	1.171	1.0178–1.273^*∗*^	—	—
NEUT#	1.168	1.074–1.271^*∗*^	—	—
LY-X	0.803	0.696–0.928^*∗*^	—	—
Leukocyte parameters on discharged				
WBC	1.541	1.240–1.194^*∗*^	1.721	1.236–2.396^*∗*^
NEUT#	1.597	1.253–2.035^*∗*^	—	—
LY-X	1.259	1.025–1.546^*∗*^	1.476	1.028–2.118^*∗*^

Abbreviations: HR, hazard ratio; CI, confidence interval; ^*∗*^*P* < 0.05.

**Table 8:** Predictive value of leukocyte parameters for clinical outcome in patients with sepsis.

Leukocyte parameters	AUC (95% CI)	Cut-off	Se (%)	Sq (%)	PPV (%)	NPV (%)
WBC on discharged	0.900 (0.714–1.000)	10.26	90.00	100.00	100.00	90.91
LY-X on discharged	0.743 (0.579–0.907)	81.10	80.00	77.10	77.75	79.40
PCT on admission	0.581 (0.392–0.771)	0.59	91.00	78.50	80.89	89.71
PCT discharged	0.693 (0.452–0.933)	0.25	70.00	74.30	73.15	71.24
WBC + LY-X on discharged	0.900 (0.714–1.000)	0.580	90.00	100.00	100.00	90.91

Abbreviations: AUC, area under the receiver operating characteristic curve; CI, confidence interval; Se, sensitivity, Sp, specificity; PPV, positive pretest value; NPV, negative pretest value.

## Data Availability

The data that support the findings of this study are available from the corresponding author upon reasonable request.
